# Zonisamide Induces Crystalluria without Urinary pH Changes in Children and Young Adults

**DOI:** 10.1155/2013/841902

**Published:** 2013-09-19

**Authors:** Tohshin Go

**Affiliations:** Kyoto Unit Center for Japan Environment & Children's Study, Kyoto University, Graduate School of Medicine, Yoshida-Konoe-cho, Sakyo-ku, Kyoto 606-8501, Japan

## Abstract

*Purpose*. Adjunctive zonisamide therapy was demonstrated to be beneficial for multiple-disabled patients with refractory childhood-onset epilepsy. Zonisamide is well tolerated, but urolithiasis and calcium sludge in the bladder were sometimes described in patients treated with antiepileptic drug polytherapy including zonisamide. In previous studies, alkaline urine and crystalluria were shown to be risk factors for urolithiasis. Therefore, the effects of zonisamide addition and withdrawal on the urinary pH and crystalluria were investigated in patients treated with antiepileptic drug polytherapy to clarify the cause of urolithiasis induced by zonisamide. *Methods*. The urinary pH and the degree of crystalluria were retrospectively studied in epilepsy patients one month after the addition or withdrawal of zonisamide as part of their antiepileptic drug treatment regimen over the previous three years. *Results*. A total of 27 zonisamide-on patients and 16 zonisamide-off patients were enrolled in the study. The urinary pH did not change after the addition or withdrawal of zonisamide. However, the degree of crystalluria significantly increased after the addition (*P* < 0.001) of zonisamide and decreased after its withdrawal (*P* < 0.01). *Conclusions*. Zonisamide induces crystalluria without alkalinization of the urine. Crystalluria should be carefully monitored in patients treated with zonisamide to prevent urolithiasis.

## 1. Introduction

The long-term use of adjunctive zonisamide therapy was demonstrated to be beneficial for treating mentally retarded and multiple-disabled patients with highly refractory childhood-onset epilepsy [1]. Zonisamide is well tolerated, and side effects are usually mild [1]. However, urolithiasis and calcium sludge in the bladder were sometimes described in patients treated with antiepileptic drug polytherapy including zonisamide [2, 3]. In previous studies, alkaline urine and crystalluria were shown to be risk factors for urolithiasis [4–7]. Therefore, the effects of zonisamide addition and withdrawal on the urinary pH and crystalluria were investigated in patients treated with antiepileptic drug polytherapy to clarify the cause of urolithiasis induced by zonisamide.

## 2. Patients and Methods

The urinary pH and the degree of crystalluria were retrospectively studied in epilepsy patients one month after the addition or withdrawal of zonisamide as part of their antiepileptic drug treatment regimen during the previous three years. The urinary pH was examined using a test paper containing methyl red and bromothymol blue. The urinary sediments were also inspected by microscopy. The degree of crystalluria was graded as 1 (1–4/HPF), 2 (5–9/HPF), or 3 (≥10/HPF) according to the presence of crystalluria per high power field (HPF).

Patients with pyuria, bacteriuria, hematuria, or proteinuria were excluded from the study. Serum electrolytes, creatinine, urea nitrogen, and zonisamide concentration were usually measured at the same time. Urinalysis and blood examinations were all performed as a routine checkup. Urine from patients with abnormal serum electrolytes, creatinine, or urea nitrogen was not included in this study. Zonisamide was prescribed at the normal recommended dosage. None of the patients included in this study had any dysmorphic features suggestive of systemic anomalies or kidney diseases including renal tubular acidosis. Statistical analysis was performed using Wilcoxon signed-rank test. Informed consent was obtained from the caregivers of patients.

## 3. Results

**Figure 1:**
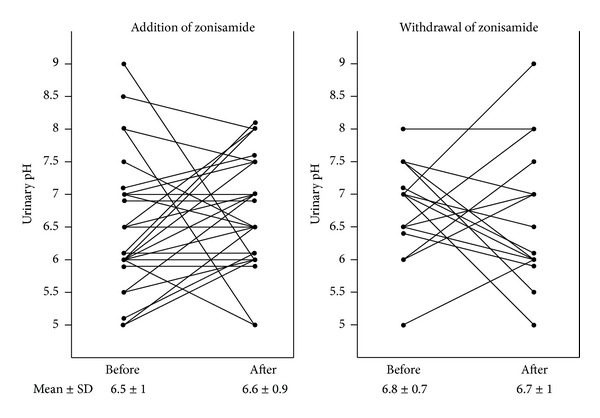
The urinary pH did not change one month after the addition or withdrawal of zonisamide from the antiepileptic drug treatment regimen.

**Figure 2:**
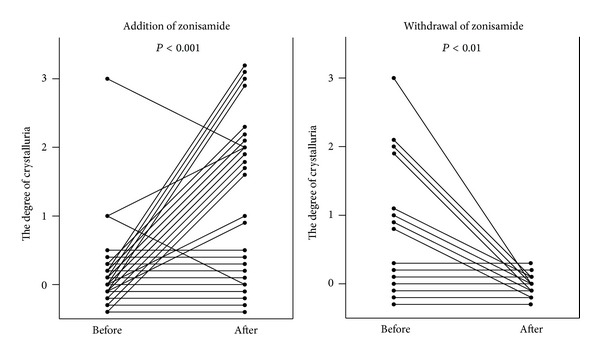
The degree of crystalluria significantly increased one month after the addition of zonisamide (*P* < 0.001) and decreased one month after its withdrawal (*P* < 0.01).

A total of 27 zonisamide-on patients aged from 1 to 25 years (10.1 ± 1.1 years, mean ± standard error), comprising 15 males and 12 females, and 16 zonisamide-off patients aged from 2 to 21 years (11.4 ± 1.3 years), consisting of 10 males and 6 females, were enrolled in this study. Serum zonisamide concentration one month after the addition of zonisamide was 17.4 ± 1.4 *μ*g/mL (mean ± standard error) and one month before the withdrawal of zonisamide was 13.6 ± 2.6 *μ*g/mL. Classification of epilepsies in patients included in this study is described in Table 1. The urinary pH did not change after the addition or withdrawal of zonisamide from the antiepileptic drug treatment regimen (Figure 1). However, the degree of crystalluria significantly increased after the addition of zonisamide (Figure 2, *P* < 0.001) and decreased after its withdrawal (Figure 2, *P* < 0.01). 

## 4. Discussion

The present study clearly indicates that zonisamide induces crystalluria, which is consistent with previous studies demonstrating that the frequency of crystalluria is higher in patients treated with zonisamide either as monotherapy or polytherapy [4, 5]. In addition, crystalluria was induced by zonisamide addition and resolved by its withdrawal without urinary pH changes in this study, demonstrating that a change in the urinary pH was not the cause of crystalluria. Because crystalluria is one of the risk factors for urolithiasis, the present study suggests that urolithiasis induced by zonisamide does not seem to result from alkalinization of the urine. Zonisamide is a carbonic anhydrase inhibitor as well as acetazolamide. Acetazolamide-induced urolithiasis is considered to result from renal tubular acidosis and alkalinization of the urine [8]. However, the potency of zonisamide is 100 times less than that of acetazolamide in inhibiting carbonic anhydrase [9]. Therefore, the mechanism of urolithiasis associated with zonisamide seems to differ from that associated with acetazolamide. The present study supports this hypothesis. There is another possibility that zonisamide might induce alkalinization of the urine after long-time use more than one month. Further studies are necessary to clarify the mechanism of urolithiasis associated with zonisamide. 

 Crystalluria should be carefully monitored in patients treated with zonisamide to prevent urolithiasis. Zonisamide-treated patients with crystalluria should increase fluid intake to induce spontaneous regression and avoid concomitant treatment with topiramate and/or ketogenic diet and not to develop urolithiasis [10].

## Figures and Tables

**Table 1:** Classification of epilepsies of patients included in the study.

	Idiopathic localization related	Symptomatic localization related	Symptomatic generalized	Undetermined
ZNS-on patients	1	19	3	4
ZNS-off patients	0	14	2	0
